# Associations Between Night Shifts and Comorbid Depressive–Anxiety Symptoms Among Chinese Nurses: Indirect Associations via Sleep Quality and Duration

**DOI:** 10.1155/jonm/9487063

**Published:** 2026-02-24

**Authors:** Chenhao Zhang, Jiali Zhou, Yi Zhou, Siyu Zhu, Weidi Sun, Shiyi Shan, Zeyu Luo, Denan Jiang, Lili Yang, Peige Song

**Affiliations:** ^1^ Center of Clinical Big Data and Analytics of the Second Affiliated Hospital and School of Public Health, Zhejiang University School of Medicine, Hangzhou, China, zju.edu.cn; ^2^ Zhejiang Key Laboratory of Intelligent Preventive Medicine, Hangzhou, Zhejiang, China; ^3^ The Fourth Affiliated Hospital of School of Medicine, and International School of Medicine, International Institutes of Medicine, Zhejiang University, Yiwu, China, zju.edu.cn; ^4^ Department of Nursing, The Fourth Affiliated Hospital, International Institutes of Medicine, Zhejiang University School of Medicine, Yiwu, Zhejiang, China, zju.edu.cn

**Keywords:** anxiety, comorbid symptoms, depression, mediation analysis, shift scheduling, sleep quality

## Abstract

**Objectives:**

Irregular sleep, frequently resulting from night shifts, is associated with various mental health issues. However, the specific associations among these factors remain unclear. This study aimed to investigate the associations of night shift frequency with depressive, anxiety, and comorbid depressive–anxiety symptoms and to estimate the indirect associations through sleep quality and sleep duration.

**Methods:**

A cross‐sectional study was conducted among nurses in seven hospitals in Zhejiang Province in 2023. A self‐administered questionnaire was utilized to collect sociodemographic, work‐related, lifestyle, and mental health information. Multivariable logistic regression models were used to examine the associations of night shift frequency, sleep duration, sleep quality, and mental health outcomes. Additionally, we used a mediation analysis to estimate indirect effects through sleep quality and sleep duration.

**Results:**

A total of 2037 nurses were included in the study. Compared to low‐frequency night shifts, high‐frequency night shifts were significantly associated with higher odds of depressive symptoms (odds ratio [OR] = 1.46, 95% confidence interval [CI] = 1.20–1.77), anxiety symptoms (OR = 1.29, 95% CI = 1.06–1.58), and comorbid depressive–anxiety symptoms (OR = 1.34, 95% CI = 1.09–1.65). The statistical indirect association via sleep quality accounted for 19.5%, 25.9%, and 19.0% of the total association for depressive, anxiety, and comorbid depressive–anxiety symptoms, respectively. The indirect effect through sleep duration was not statistically significant.

**Conclusions:**

In this cross‐sectional study, a higher frequency of night shifts was significantly associated with poorer mental health outcomes among Chinese nurses, with sleep quality serving as a statistical mediator. These findings suggest that optimizing shift scheduling, enhancing institutional support for sleep recovery, and integrating sleep quality monitoring into occupational health policies may be effective strategies to promote nurse well‐being. This study provides empirical evidence to inform nursing management practices and health workforce policy.

## 1. Introduction

Nurses, consistently ranked among the most trusted professions [1], are particularly susceptible to occupational stress, which places them at heightened risk for mental health disorders such as depression and anxiety [2]. Globally, approximately one‐third of nurses suffer from depression, and 23.2% experience anxiety [3]. In 2023, several Chinese studies indicated that 31.9% of nurses experienced depression [4] and 65.1% suffered from anxiety [4, 5]. These mental health challenges are linked to impaired job performance, increased absenteeism, higher turnover rates, and ultimately a reduced overall quality of patient care [6]. The demanding nature of nursing, including extra shifts, frequent night shifts, and skipped breaks, exacerbates this vulnerability [2]. A national report from China revealed that 74.2% of nurses regularly engaged in night shifts [7], emphasizing the need to explore the association between shift work and mental health among Chinese nurses.

Night shifts, in particular, have been identified as a significant risk factor for various mental health issues [8–10]. One potential mechanism underlying this association is the disruption of the sleep–wake cycle (circadian rhythm) caused by night shifts [11, 12]. Both sleep quality and duration are critical for mental health, with numerous studies highlighting their importance [13–15]. For instance, good sleep quality has been shown to protect against the development of mental health symptoms, especially depression and anxiety [16–18]. However, sleep duration presents a double‐edged sword for mental health, as both insufficient and excessive sleep duration can exacerbate depression and anxiety [14, 19, 20]. Night shifts are associated with disrupted sleep patterns and poorer sleep quality [12, 21], as well as with increased risk of mental health issues [22]. Despite this evidence, the respective roles that sleep quality and duration play as potential indirect pathway variables in the relationship between night shifts and mental health outcomes remain insufficiently understood.

Nurses are uniquely exposed to a combination of psychosocial and organizational stressors that can intensify the effects of night shift work on sleep and mental health [23]. The nursing work environment is characterized by high patient acuity, unpredictable emergencies, and the need for sustained vigilance, all factors linked to shift work disorder and increased psychological burden [24]. These conditions often arise under insufficient staffing and limited recovery time between shifts, factors shown to exacerbate fatigue and emotional stress [25]. For example, Chinese nurses face complex work environments that contribute to burnout and job dissatisfaction [26, 27]. Beyond individual‐level circadian disruptions, the broader organizational and psychosocial context of nursing practice may further amplify the mental health risks associated with night shifts. In the Chinese context, the hierarchical structure of hospitals, rigid scheduling systems, and high nurse‐to‐patient ratios further intensify the negative impacts of night shift work, making it especially important to investigate how shift‐related factors influence mental health in this occupational group [28–30].

The intense night shift schedules endured by Chinese nurses during the COVID‐19 pandemic have brought the mental health consequences of such work patterns into sharper public focus [31]. Although numerous studies have investigated the impact of night shifts on depression and anxiety [32–34], most of them have focused on workers, police officers, or healthcare workers in intensive care units [12, 33, 35], with relatively few addressing Chinese nurses specifically. Moreover, most previous studies have considered anxiety or depression independently [10, 31, 33], overlooking their frequent comorbidity. Anxiety frequently co‐occurs with depression [36], with approximately 39% of individuals with a generalized anxiety disorder (GAD) also meeting the diagnosis criteria of depression [37], and about two‐thirds of individuals with depression experiencing anxiety [38–40]. This comorbidity is associated with more severe symptoms and greater impairment in mental health and daily functioning [19]. Therefore, studies on anxiety and depression should also account for the co‐occurrence of symptoms to fully understand their combined effects. These findings underscore the importance of understanding how occupational structures and scheduling policies may contribute to psychological vulnerability in frontline healthcare workers.

To address these gaps, the present study made three distinct contributions to the existing literature. First, while much of the evidence comes from Western healthcare systems or general working populations, multicenter evidence quantifying night shift frequency in Chinese nurses remains limited. Chinese nurses work in a context characterized by high patient‐to‐nurse ratios [28, 29] and limited autonomy in shift scheduling [30], with constrained recovery time under chronic understaffing [25]. These contextual factors may shape the association between night shift work and mental health in ways that warrant dedicated investigation. Second, whereas prior studies often examined depression or anxiety separately [10, 31, 33], we assess the co‐occurrence of depressive and anxiety symptoms, which has been associated with greater functional impairment and poorer outcomes [19, 36] but has received comparatively less attention in occupational health research on nurses. Third, we evaluate sleep quality and sleep duration simultaneously as potential indirect pathways linking night shifts to mental health outcomes. Although sleep‐related mechanisms have been discussed [10, 33, 34], many studies focused on sleep duration alone or used composite sleep indicators without distinguishing sleep quality from quantity, an important distinction for understanding possible targets for workplace health strategies.

Accordingly, we asked: (1) To what extent is night shift frequency associated with depressive, anxiety, and comorbid depressive–anxiety symptoms among Chinese nurses? (2) Do sleep quality and sleep duration independently account for these associations? We hypothesized that higher night shift frequency would be associated with elevated mental health symptoms and that the indirect pathway through sleep quality would be stronger than that through sleep duration.

## 2. Methods

### 2.1. Study Design and Participants

The cross‐sectional survey was conducted in seven hospitals in Zhejiang Province, China, from March to April 2023 using an online survey platform. The participating hospitals were distributed across seven cities in Zhejiang Province: Taizhou (*n* = 1,218, 59.8%), Lishui (*n* = 254, 12.5%), Jinhua (*n* = 207, 10.2%), Ningbo (*n* = 163, 8.0%), Quzhou (*n* = 96, 4.7%), Hangzhou (*n* = 73, 3.6%), and Shaoxing (*n* = 26, 1.3%). The majority of participants (*n* = 1,963, 96.4%) worked in hospital settings, with smaller numbers from primary care facilities (1.8%), public health institutions (1.5%), and other healthcare organizations (0.3%).

All participants were required to read and electronically sign an informed consent form prior to beginning the questionnaire. The survey system was programmed to prevent participation unless consent was provided. Only nurses who signed the informed consent form and were selected through convenience sampling were eligible. Electronic informed consent was obtained from all participants prior to participation. Individuals who were interns, nursing students, or who withdrew before finishing the survey were excluded. This study was approved by the Ethical Committee of Zhejiang University School of Medicine (No. ZGL202302–02), and all procedures were conducted in accordance with the Declaration of Helsinki.

**FIGURE 1:**
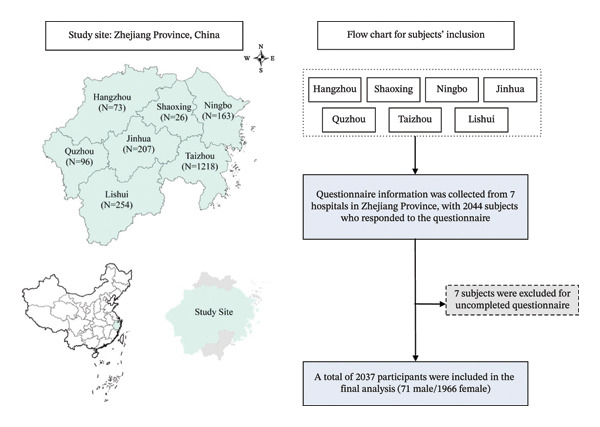
Flowchart for selecting subjects in this study.

(1) n=Z12−α/2·ρ1−ρδ2,

A sample size was calculated using the standard formula for estimating proportions in epidemiological surveys as follows:where $Z_{12-\alpha /}^{2}$ = 1.96, corresponding to a 95% confidence level; *p* = 0.418 was the expected prevalence of mental health symptoms among nurses based on previous research [4, 5]; and *δ* = 0.1 was the allowable error. The minimum required sample size should be approximately 535. To account for potential nonresponses and invalid data, we increased the sample size by 20%, resulting in a final target of 642 participants. Eventually, the initial study population included 2044 nurses. Seven individuals who did not complete the questionnaires before the deadline were excluded. Finally, data from 2037 participants were included in the analysis (Figure 1 and Table S1).

### 2.2. Instruments

Information for this study was collected through an online structured questionnaire (Questionnaire S1), administered by the nursing directors at each hospital. The main sections included sociodemographic, lifestyle, work‐related, and mental health information.

### 2.3. Sociodemographic Factors

Sociodemographic factors included age, sex, residence, education level, and monthly income. The sociodemographic section of the questionnaire was adapted from the China Health and Nutrition Survey (CHNS) [41], and the standard epidemiological indicators outlined in the Chinese academic textbook “Epidemiology” [42].

### 2.4. Lifestyle Factors

Lifestyle factors considered were smoking status, drinking status, sleep quality, and sleep duration. Sleep quality was assessed using a single‐item sleep quality scale (SQS), which asked participants to rate their overall/typical sleep quality on a scale from 0 (“terrible”) to 10 (“excellent”) [43]. This tool has been psychometrically validated, showing strong validity with established instruments (Pittsburgh Sleep Quality Index: *r* = −0.92) and acceptable test–retest reliability (ICC = 0.74) [43]. The SQS was selected for its brevity and prior validation to reduce respondent burden in an online survey. The sleep duration was self‐reported by participants. The question was designed based on the National Health and Nutrition Examination Survey [44]: “How much sleep do you usually get (hours)?” In our survey, the question was designed as follows: “How much sleep do you get each night?” Sleep duration was categorized as < 7 h or ≥ 7 h. This cutoff point was selected based on the general recommendation for adults to sleep at least 7 h each day for optimal health [45, 46]. While excessively long sleep duration (> 9 h) has also been associated with adverse mental health outcomes [45], our data did not include a sufficient number of participants sleeping ≥ 9 h to form a separate group. Therefore, we focused on the commonly used threshold of 7 h to distinguish inadequate sleep.

### 2.5. Work‐Related Factors

Following a previous study [47], night shift frequency was measured by assessing the number of night shifts nurses worked each month. In our study, night shift was defined as a shift that occurred between 22:00 and 7:00 the next day. This definition aligns with previous reports on shift work among nurses and reflects the standard scheduling practice in most Chinese hospitals [48]. A self‐reported question was used in the survey, asking the participants: “How many night shifts did you work in the past month?” (see Table 1 for distribution). Night shift frequency was categorized into three groups (≤ 4, 5–9, and ≥ 10 times per month) based on three considerations. First, previous research has suggested that working ≤ 4 night shifts per month may represent a threshold for maintaining well‐being among nurses, with higher frequencies associated with elevated health risks [49]. Second, examination of our sample distribution showed that these categories captured meaningful variation, with approximately 50.2% working ≤ 4 shifts, 36.9% working 5–9 shifts, and 12.9% working ≥ 10 shifts per month (see Table 1), ensuring sufficient numbers in each category for stable estimation and comparisons. Third, these categories reflect common scheduling practices in many Chinese hospitals [7], where nurses typically rotate through varying frequencies of night shifts depending on staffing needs and departmental policies [48]. The three‐category structure allowed us to examine potential dose–response patterns while maintaining interpretability and clinical relevance. While validated scales exist for measuring job stress, our study focused on the quantifiable exposure of night shift frequency, which has been consistently linked to sleep disturbances and mental health outcomes. A job stress scale was not included to reduce respondent burden and keep the questionnaire concise.

**TABLE 1:** Distribution of depressive, anxiety, and comorbid depressive–anxiety symptoms.

Variables	Depressive symptoms			Anxiety symptoms			Comorbid depressive–anxiety symptoms		
	No (*n* = 971)	Yes (*n* = 1066)	*p*	No (*n* = 715)	Yes (*n* = 1322)	*p*	No (*n* = 1059)	Yes (*n* = 978)	*p*
Age, years^a^	32.00 (26.00–38.00)	32.00 (26.00–37.00)	0.214	33.00 (26.00–38.00)	32.00 (26.00–37.00)	**0.031**	32.00 (26.00–38.00)	31.00 (25.25–37.00)	0.096
Sex			0.874			0.365			0.921
Male	35 (3.6)	36 (3.4)		29 (4.1)	42 (3.2)		36 (3.4)	35 (3.6)	
Female	936 (96.4)	1030 (96.6)		686 (95.9)	1280 (96.8)		1023 (96.6)	943 (96.4)	
Residence			0.335			0.156			0.232
Rural	468 (48.2)	490 (46.0)		352 (49.2)	606 (45.8)		512 (48.3)	446 (45.6)	
Urban	503 (51.8)	576 (54.0)		363 (50.8)	716 (54.2)		547 (51.7)	532 (54.4)	
Education level			0.352			0.376			0.874
Bachelor’s degree below	218 (22.5)	259 (24.3)		176 (24.6)	301 (22.8)		250 (23.6)	227 (23.2)	
Bachelor’s degree or above	753 (77.5)	807 (75.7)		539 (75.4)	1021 (77.2)		809 (76.4)	751 (76.8)	
Monthly income			**< 0.001**			0.133			**0.001**
< CNY 4000	91 (9.4)	146 (13.7)		72 (10.5)	162 (12.3)		101 (9.5)	136 (13.9)	
CNY 4000–6000	362 (37.3)	391 (36.7)		269 (37.6)	484 (36.6)		396 (37.4)	357 (36.5)	
CNY 6000–8000	277 (28.5)	335 (31.4)		199 (27.8)	413 (31.2)		306 (28.9)	306 (31.3)	
CNY 8000–10000	151 (15.6)	133 (12.5)		114 (15.9)	170 (12.9)		165 (15.6)	119 (12.2)	
≥ CNY 10000	90 (9.3)	61 (5.7)		58 (8.1)	93 (7.0)		91 (8.6)	60 (6.1)	
Frequency of night shift (per month)			**< 0.001**			**0.027**			**0.002**
≤ 4 times	528 (54.4)	495 (46.4)		383 (53.6)	640 (48.4)		567 (53.5)	456 (46.6)	
5–9 times	342 (35.2)	409 (38.4)		256 (35.8)	495 (37.4)		375 (35.4)	376 (38.4)	
≥ 10 times	101 (10.4)	162 (15.2)		76 (10.6)	187 (14.1)		117 (11.0)	146 (14.9)	
Smoking status			0.876			0.908			0.547
No	960 (98.9)	1052 (98.7)		707 (98.9)	1305 (98.7)		1048 (99.0)	964 (98.6)	
Yes	11 (1.1)	14 (1.3)		8 (1.1)	17 (1.3)		11 (1.0)	14 (1.4)	
Drinking status			**0.004**			**0.009**			**0.003**
No	926 (95.4)	982 (92.1)		684 (95.7)	1224 (92.6)		1009 (95.3)	899 (91.9)	
Yes	45 (4.6)	84 (7.9)		31 (4.3)	98 (7.4)		50 (4.7)	79 (8.1)	
Sleep duration			**< 0.001**			**< 0.001**			**< 0.001**
≥ 7 h	496 (51.1)	273 (25.6)		379 (53.0)	390 (29.5)		523 (49.4)	246 (25.2)	
< 7 h	475 (48.9)	793 (74.4)		336 (47.0)	932 (71.5)		536 (50.6)	732 (74.8)	
Sleep quality			**< 0.001**			**< 0.001**			**< 0.001**
Good	382 (39.3)	130 (12.2)		296 (41.4)	216 (16.3)		393 (37.1)	119 (12.2)	
Fair	468 (48.2)	522 (49.0)		324 (45.3)	666 (50.4)		510 (48.2)	480 (49.1)	
Poor	121 (12.5)	414 (38.8)		95 (13.3)	440 (33.3)		156 (14.7)	379 (38.8)	

*Note:* Bold values indicate statistical significance at *p* < 0.05.

^a^Values were presented as medians with interquartile range.

### 2.6. Mental Health Information

Depressive and anxiety symptoms were assessed using the 10‐item Center for Epidemiologic Studies‐Depression Scale (CESD‐10) [50] and the Generalized Anxiety Disorder 7‐item Scale (GAD‐7) [51].

The CESD‐10 is a brief screening instrument designed to measure depressive symptomatology in the general population. The Chinese version of the CESD‐10 used in this study was adapted from the version employed in the China Health and Retirement Longitudinal Study (CHARLS) [52]. The scale consists of 10 items assessing various aspects of depression experienced during the past week. Each item is rated on a four‐point Likert scale: 0 = “Rarely or none of the time (less than 1 day),” 1 = “Some or a little of the time (1‐2 days)”, 2 = “Occasionally or a moderate amount of time (3‐4 days)”, and 3 = “Most or all of the time (5–7 days)”. Total scores range from 0 to 30, with higher scores indicating more severe depressive symptoms. Following established practice [52], a cutoff score of ≥ 10 was used to identify depressive symptoms. In the current sample, the CESD‐10 demonstrated good internal consistency (Cronbach’s *α* = 0.815).

The GAD‐7 is a validated screening tool for GAD in research. The Chinese version was translated and validated by He et al. [53]. The scale consists of 7 items assessing anxiety symptoms over the past 2 weeks. Each item is rated on a four‐point Likert scale: 0 = “Not at all,” 1 = “Several days,” 2 = “More than half the days,” and 3 = “Nearly every day.” Total scores range from 0 to 21, with higher scores indicating more severe anxiety symptoms. A cutoff score of ≥ 5 was used to indicate the presence of anxiety symptoms, which has been validated in Chinese populations [53]. In the current sample, the GAD‐7 demonstrated excellent internal consistency (Cronbach’s *α* = 0.898).

The comorbid depressive–anxiety symptoms were defined as the presence of both depressive and anxiety symptoms simultaneously [54]. Participants were categorized into four mutually exclusive groups: neither symptom, depressive‐only, anxiety‐only, and comorbid symptoms.

### 2.7. Statistical Analysis

The continuous variables were displayed as the median and interquartile range (IQR) due to skewness in normality tests. Results were presented as numbers (*n*) with percentages (%) for categorical variables. Kruskal–Wallis tests were used to compare the age and depressive anxiety and comorbid depressive–anxiety symptoms. For categorical variables, a chi‐square (*χ* ^2^) test was conducted.

The multivariable logistic regression models were conducted to explore the associations between night shift frequency, depressive, anxiety, and comorbid depressive–anxiety symptoms. Covariates were selected based on previous studies [55]. We considered sociodemographic factors (age, sex, education level, monthly income, and residence) and lifestyle factors (smoking and drinking status) as possible confounders. Model a was adjusted for age, sex, residence, education level, monthly income, smoking and drinking status, sleep quality, and duration. Model b was adjusted by age, sex, residence, education level, monthly income, smoking and drinking status, and sleep quality. Model c was adjusted for age, sex, residence, education level, monthly income, smoking and drinking status, and sleep duration. Multicollinearity was assessed by calculating variance inflation factors (VIFs) for all predictor variables. The VIF values for sleep quality and sleep duration were both about 1.12–1.13, indicating no significant multicollinearity. As a sensitivity analysis, we estimated prevalence ratios (PRs) to assess whether odds ratios (ORs) overestimated association magnitudes given the high prevalence of mental health outcomes in our sample (depressive symptoms: 52.3%; anxiety symptoms: 64.9%; comorbid depressive–anxiety symptoms: 48.0%). We used modified Poisson regression with robust variance estimation, as log‐binomial regression models did not converge due to the binary exposure and high outcome prevalences. The Poisson models used the same exposure variable (night shift frequency > 4 vs. ≤ 4 times per month), outcomes (depressive, anxiety, and comorbid depressive–anxiety symptoms), and covariate adjustments (age, sex, residence, education level, monthly income, smoking status, drinking status, sleep quality, and sleep duration) as Model a in the main logistic regression analyses.

Mediation analyses were conducted to estimate indirect effects of night shift frequency on outcomes through sleep quality and sleep duration. The analyses were conducted using the PROCESS procedure implemented in the bruceR package [56] in R, following the framework described by Hayes (2018) [57]. Night shift frequency was treated as a binary categorical exposure (≤ 4 vs. > 4 times/month) and entered into the models as a factor (reference = ≤ 4 times/month), consistent with prior work recommending ≤ 4 night shifts per month for optimal well‐being [49]. Additionally, sleep quality was dichotomized into poor‐to‐fair (SQS score 0–6) and good (SQS score 7–10) based on the validated approach by previous research [43]. Because both mediators and outcomes were binary, all paths were estimated using logistic regression and reported as unstandardized coefficients (*B*) on the log‐odds scale. Indirect effects were estimated using the product‐of‐coefficients approach and evaluated using bias‐corrected bootstrap 95% confidence intervals based on 2000 resamples; indirect effects were considered statistically significant when the bootstrap confidence interval did not include zero. The proportion mediated was reported descriptively as the indirect effect/total effect on the logit scale. All mediation models adjusted for age, sex, residence, education level, monthly income, smoking status, drinking status, and either sleep duration (when sleep quality was the mediator) or sleep quality (when sleep duration was the mediator). Exposure–mediator interaction terms were not included. This analysis used a regression‐based (non‐counterfactual) framework; given the cross‐sectional design, the indirect effects are interpreted as statistical indirect associations rather than causal (natural) indirect effects. We conducted sensitivity analyses additionally adjusting for professional title and years of nursing experience, and further adjusting for department assignment.

All data were analyzed in R Version 4.3.1. A two‐sided *p-*value of less than 0.05 indicated statistical significance.

## 3. Results

### 3.1. Characteristics of Study Participants

A total of 2037 nurses participated in this study, with a response rate of 99.7% (2037/2044). The participants were predominantly female (96.5%, *n* = 1966) with a median age of 32 years (IQR: 26–37 years). The majority had a Bachelor’s degree or above (76.6%), were nonsmokers (98.8%), and did not consume alcohol (93.7%). Regarding sleep patterns, 62.2% (*n* = 1268) reported sleeping less than 7 h per night, and 74.9% (*n* = 1525) reported fair‐to‐poor sleep quality. Concerning mental health outcomes, 52.3% (*n* = 1066) experienced depressive symptoms, 64.9% (*n* = 1322) experienced anxiety symptoms, and 48.0% (*n* = 978) experienced comorbid depressive–anxiety symptoms. Regarding night shift frequency, 50.2% (*n* = 1023) worked ≤ 4 night shifts per month, 36.9% (*n* = 751) worked 5–9 shifts, and 12.9% (*n* = 263) worked ≥ 10 shifts per month. Examining symptom patterns showed substantial overlap between depressive and anxiety symptoms. Among the 2037 participants, 627 (30.8%) screened positive for neither depressive nor anxiety symptoms, 88 (4.3%) screened positive for depressive symptoms only, 344 (16.9%) screened positive for anxiety symptoms only, and 978 (48.0%) screened positive for both depressive and anxiety symptoms (comorbid depressive–anxiety symptoms). Comorbidity was the most common pattern, affecting nearly half of the sample. Among nurses who screened positive for depressive symptoms (*n* = 1066), most (91.7%, 978/1066) also screened positive for anxiety symptoms. Similarly, among those who screened positive for anxiety symptoms (*n* = 1322), a large proportion (74.0%, 978/1322) also screened positive for depressive symptoms. These results highlight the substantial co‐occurrence of depressive and anxiety symptoms in this nursing population. Detailed sociodemographic characteristics are presented in Table 1.

### 3.2. Associations of Night Shift Frequency, Sleep Quality and Duration, and Mental Health

The results indicated the associations between night shift frequency, sleep quality and duration, and mental health. Nurses with a high frequency of night shift (OR: 1.76, 95% CI = 1.31–2.38) and poor sleep quality (OR = 7.05, 95% CI = 5.20–9.62) had higher odds of experiencing depressive symptoms. Nurses with long sleep duration (OR = 0.56, 95% CI = 0.46–0.70) had lower odds of suffering from anxiety symptoms. Additionally, high frequency of night shift (OR = 1.48, 95% CI 1.08–2.03) and poor sleep quality (OR = 4.58, 95% CI = 3.37–6.26) increased the odds of experiencing anxiety symptoms, while longer sleep duration (OR = 0.58, 95% CI = 0.47–0.71) decreased the odds of anxiety symptoms. On the other side, high odds of comorbid depressive–anxiety symptoms were associated with night shifts over ten times (OR: 1.58, 95% CI = 1.18–2.11) and poor sleep quality (OR = 5.71, 95% CI = 4.24–7.74) in nurses, while longer sleep duration (OR = 0.56, 95% CI = 0.46–0.70) was associated with lower odds of comorbid depressive–anxiety symptoms (Table S2). In addition, nurses with high night frequency had higher odds of experiencing poor sleep quality (OR = 2.13, 95% CI = 1.46–3.17). However, there was no significant association between night shift frequency and sleep duration (Table S3). The associations between night shift frequency and depressive, anxiety, and comorbid depressive–anxiety symptoms remained materially unchanged in sensitivity analyses adjusting for professional title, work years, and department assignment (Table S4). Sensitivity analyses using PRs confirmed the associations between night shift frequency and mental health outcomes while showing smaller effect magnitudes, as expected when outcomes are common. For night shift frequency > 4 versus ≤ 4 times per month, the PRs were 1.14 (95% CI = 1.05–1.24) for depressive symptoms, 1.07 (95% CI = 1.00–1.14) for anxiety symptoms, and 1.14 (95% CI = 1.04–1.25) for comorbid depressive–anxiety symptoms (Table S5). While these PRs were considerably smaller than the corresponding ORs for the highest night shift frequency category reported in Table S2 (ORs: 1.76, 1.48, and 1.58, respectively), all associations remained statistically significant, confirming that the main conclusions are robust across different effect measures.

### 3.3. Mediation Effects

**FIGURE 2:**
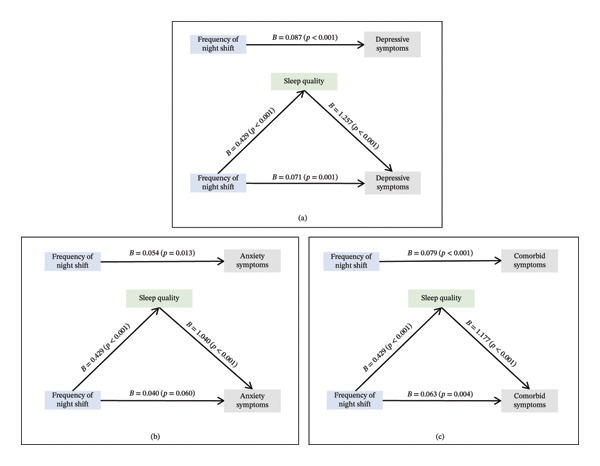
Mediation pathways showing associations of night shift frequency with depressive, anxiety, and comorbid depressive–anxiety symptoms through sleep quality. Note: Coefficients are logistic regression coefficients (*B*) on the log‐odds scale. Indirect effects and 95% CIs are bias‐corrected bootstrap CIs (2000 resamples). All models were adjusted for age, sex, residence, education, monthly income, and sleep duration. Indirect effects are interpreted as statistical indirect associations.

Figure 2 summarizes the indirect‐effect models examining whether sleep quality may explain part of the association between night shift frequency and mental health outcomes. For depressive symptoms, night shift frequency showed a significant total association (*B* = 0.087, 95% CI = 0.046–0.133), and the indirect effect via sleep quality was significant (indirect effect *B* = 0.017, 95% bootstrap CI = 0.007–0.033). For anxiety symptoms, the total association was significant (*B* = 0.054, 95% CI = 0.014–0.101), with a significant indirect effect via sleep quality (*B* = 0.014, 95% bootstrap CI = 0.006–0.029); the direct effect was attenuated and not statistically significant after including sleep quality (*B* = 0.04, 95% CI = −0.002–0.082). For comorbid depressive–anxiety symptoms, the total association was significant (*B* = 0.079, 95% CI = 0.037–0.124), and the indirect effect via sleep quality was significant (*B* = 0.015, 95% bootstrap CI = 0.007–0.031). All models adjusted for age, sex, residence, education, monthly income, smoking status, drinking status, and sleep duration. These indirect effects should be interpreted as statistical associations rather than causal mediation, because the cross‐sectional design does not establish temporal ordering among night shifts, sleep quality, and mental health symptoms. Overall, the indirect effect through sleep quality accounted for 19.5%, 25.9%, and 19.0% of the total association between night shift frequency and depressive, anxiety, and comorbid depressive–anxiety symptoms, respectively (see Figure 2 for indirect‐effect estimates and 95% CIs).

## 4. Discussion

This multicenter study contributes to the literature on night shift work and mental health among Chinese nurses in three ways. First, we observed positive associations between higher night shift frequency and depressive, anxiety, and comorbid depressive–anxiety symptoms (ORs ranging from 1.48 to 1.76). Although prior meta‐analytic evidence based largely on Western samples has reported comparable associations for night shifts and depressive symptoms (pooled OR = 1.33) [34], the high prevalence of symptoms observed in our sample highlights the potential mental health burden in this occupational group within the Chinese context. These patterns may relate to contextual factors in Chinese nursing practice, such as high patient‐to‐nurse ratios [28, 29], and limited opportunities for recovery between shifts [25]. Second, our study addresses a gap in the occupational health literature by assessing the co‐occurrence of depressive and anxiety symptoms, where these outcomes are often examined separately [10, 31, 33]. In our sample, nearly half of the participants met criteria for both depressive and anxiety symptoms, and the association between night shift frequency and symptom co‐occurrence underscores the importance of considering comorbidity when evaluating mental health risks among nurses. Third, our mediation analysis suggests that the indirect pathway through sleep quality accounted for a meaningful proportion (19.5%–25.9%) of the association between night shift frequency and mental health outcomes, whereas the indirect pathway through sleep duration was comparatively smaller. This extends prior mediation studies that frequently operationalized sleep as duration only or used composite sleep measures, and rarely compared sleep quality and duration simultaneously, particularly in large, multicenter nurse samples [12, 13]. This finding indicates that sleep quality may be a particularly relevant target for workplace health strategies aimed at mitigating the mental health correlates of night shift work in nursing settings.

Our study found that frequent night shifts were associated with increased odds of depressive, anxiety, and comorbid depressive–anxiety symptoms, which is consistent with previous research [9, 34, 58]. Poor mental health is often observed alongside night shift‐related emergency situations, which are associated with increased pressure among nurses at night [59]. Additionally, a high frequency of night shifts is associated with reduced time for leisure activities during the day [60, 61], which may be linked to social isolation and mental health issues. Besides, previous research has primarily focused on either depression or anxiety alone, often overlooking their comorbid presentation [9, 58, 62]. In contrast, our study comprehensively examined the coexistence of depressive and anxiety symptoms, revealing a compounded risk for nurses exposed to frequent night shifts. Moreover, our findings are in line with studies among nurses from diverse geographic regions. Similar associations between night shifts and depressive symptoms, together with poorer sleep quality, have been documented among European nurses [9], Asian populations [58], and, more recently, in a study among Saudi Arabian nurses [63]. Meta‐analyses across multiple occupational groups have also confirmed these patterns [34]. However, the magnitude of associations in our study was somewhat different from that reported among general working populations [8, 34], which may in part relate to features of the Chinese nursing context, including higher patient‐to‐nurse ratios [28, 29], limited institutional support for shift work recovery [25], and cultural factors that may limit help‐seeking for mental distress [27].

The study findings demonstrated that sleep quality and duration are negatively correlated with depressive, anxiety, and comorbid depressive–anxiety symptoms. Poor sleep quality and short sleep duration are associated with sleep deprivation. Chronic sleep deprivation has been linked to alterations in mood‐regulating neurotransmitters such as serotonin and dopamine, which may be associated with mental health symptoms, as shown in other studies [64, 65]. Furthermore, while prior studies have demonstrated associations between both short and long sleep durations and mental health symptoms [14, 15, 19], our findings suggest a divergent pattern among Chinese nurses. Unlike studies focusing on general populations or other occupational groups, our research indicates that sleep duration did not significantly mediate the effects of night shift frequency on mental health outcomes among Chinese nurses. This contrasts with prior studies in general working populations [8, 9, 12], which reported significant mediation effects of short sleep duration. This discrepancy may reflect differences in study populations, sleep assessment tools, and the Chinese nursing context, where rapid shift rotations and limited recovery time [25, 30] may make sleep quality more salient than sleep duration. These findings highlight sleep quality as a particularly relevant indirect pathway variable in this occupational setting.

The research also showed that the high night shift frequency was significantly associated with poor sleep quality, consistent with previous studies [34, 66]. Firstly, high night shift frequency is associated with greater exposure to artificial light at night. Exposure to artificial light at night is associated with suppressed melatonin production, a hormone that regulates sleep [67, 68], which may impair sleep quality. Furthermore, night shift workers often experience fragmented sleep [69] and are at higher risk for various diseases, such as cardiovascular diseases [34]. The stress and concerns related to both these diseases and fragmented sleep are more likely to lead night shift nurses to report poor sleep quality [66].

While there are associations between sleep duration and mental health symptoms, it is important to note that there is no significant correlation between night shifts and sleep duration. This finding suggests that sleep duration did not emerge as a significant indirect pathway variable in the associations between night shift frequency and depressive, anxiety, or comorbid depressive–anxiety symptoms. This is consistent with previous research, indicating that sleep quality may be a more salient predictor of mental health outcomes than sleep duration [16, 19, 70]. An important additional explanation for this pattern may be the phenomenon of “recovery sleep” or “catch‐up sleep” on days off [71]. Nurses working frequent night shifts may compensate for sleep debt by sleeping longer during their off‐days, thereby maintaining similar total sleep duration compared to nurses with fewer night shifts [69, 71]. However, this compensatory sleep may remain of poor quality due to persistent circadian misalignment and the inability to fully restore normal sleep architecture after repeated circadian disruptions [11, 67, 68]. This recovery sleep phenomenon could explain why night shift frequency was not significantly associated with sleep duration but was strongly associated with poor sleep quality (Table S3), ultimately leading to the null indirect effect through sleep duration. The quality of sleep, rather than its quantity, may thus be the more proximal factor linking night shift work to mental health outcomes among nurses. There may be some mechanisms to explain this finding. First, short sleep duration may not capture the full extent of sleep disturbances experienced by night shift workers, as it does not account for the fragmentation or restfulness of sleep [13, 46]. Second, individuals might adapt to falling asleep during the daytime over time, and the high frequency of night shifts may not significantly impact nurses’ ability to adjust to a new sleep schedule. Finally, the psychological stress associated with night shifts and poor sleep quality may be more impactful than the total amount of sleep obtained.

The results suggest that sleep quality statistically mediates a portion of the associations between night shift frequency and depressive and comorbid depressive–anxiety symptoms. The statistical mediation was observed through several stages, highlighting the importance of sleep quality. First, a high frequency of night shifts was associated with an increased risk of depressive and anxiety symptoms [72]. Second, individuals with either or both anxiety and depressive symptoms were more likely to experience poor sleep quality [73]. Third, previous studies have indicated that night shifts are associated with poor sleep quality [70]. Therefore, it can be inferred that nurses with low night shift frequencies are more likely to maintain better mental health due to improved sleep quality. These findings are consistent with previous studies in European nurses’ research [9, 69], as well as meta‐analyses across occupational groups [34, 58], which showed that sleep quality plays an important mediating role between night shifts and mental health [10, 33, 34]. Sleep quality statistically mediated the association between night shift frequency and anxiety symptoms, with the direct association becoming nonsignificant after accounting for sleep quality. This suggests that sleep quality may be a key intermediate variable linking night shift frequency and anxiety symptoms. This result contrasts with some previous studies in Western countries and in South Korea [51, 52], where sleep quality partially mediated the relationship between night shifts and anxiety. This pattern may reflect both the predominantly female composition of our sample and organizational features of Chinese nursing practice (e.g., high patient‐to‐nurse ratios, rapid shift rotations) [28, 30, 74] that may increase the relevance of sleep quality in the observed indirect associations between night shifts and anxiety symptoms.

### 4.1. Implications for Nursing Practice and Health Policy

Given that our findings indicate sleep quality, rather than sleep duration, as the key statistical indirect pathway linking night shifts to mental health outcomes, interventions should prioritize strategies that directly target sleep quality improvement rather than simply extending sleep opportunities.

Individual‐Level Strategies: Nurses may benefit from targeted sleep quality enhancement techniques, including evidence‐based sleep hygiene education that emphasizes environmental optimization (light control, temperature regulation, noise reduction) and strategic timing of caffeine intake relative to sleep periods [75]. Mindfulness‐based stress reduction programs and cognitive behavioral therapy for insomnia (CBT‐I) have demonstrated efficacy in improving sleep quality among shift workers and could be offered as accessible resources [76, 77]. Peer support groups specifically focused on managing sleep challenges associated with night shift work may facilitate practical coping strategy exchange.

Hospital‐Level Interventions: Healthcare administrators should implement “forward‐rotating schedules” (day–evening–night rotation) rather than backward rotation, as forward rotation better aligns with circadian physiology and has been shown to improve sleep quality [11, 21, 78]. Hospitals should provide dedicated “quiet recovery rooms” or “nap pods” equipped with blackout curtains, temperature control, and comfortable seating to enable short restorative naps during breaks, interventions that directly target sleep depth and quality rather than duration alone [79]. Limiting consecutive night shifts (avoiding back‐to‐back assignments) and ensuring adequate recovery time (minimum 48 h between night shift blocks) can help prevent cumulative circadian disruption [21, 78]. Importantly, managers should incorporate the single‐item SQS as a routine “vital sign” assessment during regular staff check‐ins and performance reviews, as this validated, brief tool [43] enables early identification of nurses experiencing deterioration in sleep quality without imposing a significant administrative burden. When poor sleep quality is detected, managers should have clear referral pathways to occupational health services and employee assistance programs.

Policy‐Level Reforms: National nursing workforce policies should establish evidence‐based upper limits on night shift frequency, mandate institutional implementation of sleep quality monitoring programs, and provide dedicated funding for workplace sleep health interventions. Occupational health surveillance systems should routinely collect sleep quality metrics alongside mental health screening to enable early intervention [80]. These multilevel strategies can serve as practical responses to the mental health burden faced by nurses in high‐intensity shift systems, particularly in regions with high patient‐to‐nurse ratios and limited institutional support.

### 4.2. Strengths and Limitations

Our study had several strengths. It innovatively explored the statistical mediators of various sleep‐related factors in the associations between night shift frequency and mental health symptoms among nurses in China. Furthermore, it explored the complex interplay of comorbid depressive–anxiety symptoms within this population, enhancing our understanding of these relationships in the healthcare system.

Several limitations should be acknowledged. First, sample sizes varied substantially across the seven participating hospitals, with two sites contributing relatively small numbers (Shaoxing: *n* = 26, 1.3%; Hangzhou: *n* = 73, 3.6%) while one hospital (Taizhou) contributed the majority (*n* = 1,218, 59.8%). This imbalance may limit representativeness and reduce the precision of estimates for smaller sites. The convenience sampling approach may further affect generalizability to the broader nursing population. Second, the cross‐sectional design combined with differing recall periods (night shifts: past month; depressive symptoms: past week; anxiety symptoms: past 2 weeks; sleep: typical recent patterns) precludes causal inference and limits our ability to establish temporal ordering among night shifts, sleep disruption, and mental health symptoms. Third, nurses from the same hospital likely share similar work environments and scheduling practices, which may induce intracluster correlation. However, our analyses treated all observations as independent, potentially leading to underestimated standard errors and overly narrow confidence intervals. The sample imbalance across sites (ranging from 1.3% to 59.8%) further compounds this concern, as findings may disproportionately reflect the largest contributing site. Fourth, sleep quality was assessed using a validated single‐item measure (*r* = −0.92 with PSQI) [43] rather than multidimensional instruments. While this reduced respondent burden, a single‐item measure cannot distinguish between different sleep problem types (initiation, maintenance, early awakening), which may have attenuated associations and limited our ability to identify which specific sleep dimensions drive the observed relationships. Fifth, sleep duration was dichotomized at 7 h rather than using more granular categories. This may not capture U‐shaped associations with mental health documented in prior research [14, 15, 19], wherein both insufficient (< 6 h) and excessive (> 8‐9 h) sleep are associated with elevated symptoms. This dichotomization might also mask the potential adverse effects of long sleep duration, though such cases were rare in our sample. This information loss may partly explain why sleep duration did not emerge as a significant statistical mediator in our analyses, as the binary categorization cannot distinguish between normal sleep and potentially problematic long sleep duration. Sixth, our study did not measure important shift work characteristics (fixed vs. rotating schedules, consecutive night shifts, shift duration). While we collected data on professional title, work experience, and department assignments, some departments had small sample sizes (psychiatry: *n* = 27; general practice: *n* = 19), limiting adjustment capabilities. Other unmeasured work‐related variables include contract status, nurse‐to‐patient ratios, and validated job stress measures. Sensitivity analyses adjusting for professional title, work years, and department showed materially unchanged associations (Table S4), suggesting limited confounding by these proxies, though residual confounding from other unmeasured factors remains possible. Seventh, hospital identifiers were not collected in this anonymous online survey to protect participant confidentiality when reporting sensitive mental health information. Consequently, we could not account for within‐hospital clustering using multilevel models or cluster‐robust standard errors, nor could we calculate intracluster correlation coefficients. Without the ability to model the hierarchical data structure, we cannot definitively assess how clustering affects our estimates. Future longitudinal studies should incorporate comprehensive shift work assessments, multidimensional sleep measures (e.g., PSQI), hospital identifiers with appropriate data protection, aligned measurement windows, and detailed work environment variables to establish temporal relationships, properly account for clustering, and minimize residual confounding.

## 5. Conclusions

This cross‐sectional study found that frequent night shifts were significantly associated with elevated odds of depressive, anxiety, and comorbid depressive–anxiety symptoms among Chinese nurses, with the statistical indirect association via sleep quality, rather than sleep duration, accounting for a meaningful proportion of these associations. These findings highlight the particular vulnerability of nurses in high‐demand healthcare systems, where irregular schedules, high patient‐to‐nurse ratios, and limited recovery time may exacerbate sleep disturbances and mental health risks. Interventions aimed at improving sleep quality, optimizing shift schedules, and integrating occupational mental health programs into nursing workforce strategies may help mitigate the adverse effects of frequent night shifts. These findings contribute to a better understanding of the pathways through which occupational stressors, such as shift work, influence mental health in nursing populations.

**Nomenclature**
OR: Odd ratio
CI: Confidence interval
GAD: Generalized anxiety disorder
COVID‐19: Coronavirus disease 2019
CNY: China yuan
CESD: Center of Epidemiologic Studies‐Depression Scale
PHQ: Patient Health Questionnaire
IQR: Interquartile range

## Author Contributions

Peige Song designed the study. Lili Yang collected the data. Chenhao Zhang analyzed the data. Chenhao Zhang prepared the first draft. Chenhao Zhang, Jiali Zhou, Yi Zhou, Siyu Zhu, Weidi Sun, Shiyi Shan, Zeyu Luo, Denan Jiang, Lili Yang, and Peige Song critically revised the manuscript.

## Funding

No funding was received for this research.

## Disclosure

All authors were involved in revising the paper and gave final approval of the submitted versions.

## Ethics Statement

Ethics for conducting the study were approved by the Ethical Committee of Zhejiang University School of Medicine, Zhejiang, China (No. ZGL202302–02).

Informed consent was obtained from all subjects involved in the study.

## Consent

Please see the Ethics Statement.

## Conflicts of Interest

The authors declare no conflicts of interest.

## Supporting Information

Declaration of Use for CESD‐10 and GAD‐7 Scales.

Table S1. Characteristics of participants (*n* = 2037).

Table S2. Associations of frequency of night shift, sleep duration, sleep quality with depressive, anxiety, and comorbid depressive–anxiety symptoms.

Table S3. Associations of frequency of night shift with sleep duration and sleep quality.

Table S4. Sensitivity analyses: associations between higher night shift frequency and mental health outcomes after additional adjustment for work‐related factors.

Table S5. Sensitivity analysis: Prevalence ratios for associations between night shift frequency and mental health outcomes.

## Supporting information


**Supporting Information** Additional supporting information can be found online in the Supporting Information section.

## Data Availability

The data that support the findings of this study are available upon request from the corresponding author. The data are not publicly available due to privacy or ethical restrictions.
