# Supplementary material for: Associations Between Night Shifts and Comorbid Depressive–Anxiety Symptoms Among Chinese Nurses: Indirect Associations via Sleep Quality and Duration
Source: J Nurs Manag. 2026 Feb 24;2026:9487063. doi: 10.1155/jonm/9487063 (PMC12932965; doi:10.1155/jonm/9487063)
Supplement: Supplementary file 1 — Supporting Information Additional supporting information can be found online in the Supporting Information section. [file JONM-2026-9487063-s001.docx]

**Legends**

Declaration of Use for CESD-10 and GAD-7 Scales

Table S1. Characteristics of participants (n=2037)

Table S2. Associations of frequency of night shift, sleep duration, sleep quality with depressive, anxiety, and comorbid symptoms

Table S3. Associations of frequency of night shift with sleep duration and sleep quality

Table S4. Sensitivity analyses: associations between higher night-shift frequency and mental health outcomes after additional adjustment for work-related factors

Table S5. Sensitivity analysis: Prevalence ratios for associations between night shift frequency and mental health outcomes

**Declaration of Use for CESD-10 and GAD-7 Scales**

The Chinese version of the CESD-10 used in this study was adapted from the version employed in the China Health and Retirement Longitudinal Study (CHARLS), which is publicly available and has been widely used in academic research.

The Chinese version of GAD-7 used in this study is based on the validated translation by He’s team in 2010, which is also freely available for academic use.

Therefore, no special permission was required to use or reproduce either scale in this study.

**Table S1. Characteristics of participants (n=2037)**

| Characteristic | | Number | Percent (%) |
| --- | --- | --- | --- |
| Age, years ^a^ | | 32.00 (26.00–37.00) |  |
| Sex | |  |  |
|  | Male | 71 | 3.5 |
|  | Female | 1966 | 96.5 |
| Residence | |  |  |
|  | Rural | 958 | 47.0 |
|  | Urban | 1079 | 53.0 |
| Education level | |  |  |
|  | Bachelor’s degree below | 477 | 23.4 |
|  | Bachelor’s degree or above | 1560 | 76.6 |
| Frequency of night shift (per month) | |  |  |
|  | ≤4 times | 1023 | 50.2 |
|  | 5–9 times | 751 | 36.9 |
|  | ≥10 times | 263 | 12.9 |
| Smoking status | |  |  |
|  | No | 2012 | 98.8 |
|  | Yes | 25 | 1.2 |
| Monthly income | |  |  |
|  | <CNY 4000 | 237 | 11.6 |
|  | CNY 4000–6000 | 753 | 37.0 |
|  | CNY 6000–8000 | 612 | 30.0 |
|  | CNY 8000–10000 | 284 | 13.9 |
|  | ≥CNY 10000 | 151 | 7.5 |
| Drinking status | |  |  |
|  | No | 1908 | 93.7 |
|  | Yes | 129 | 6.3 |
| Sleep duration | |  |  |
|  | ≥7 hours | 769 | 37.8 |
|  | <7 hours | 1268 | 62.2 |
| Sleep quality | |  |  |
|  | Good | 512 | 25.1 |
|  | Fair | 990 | 48.6 |
|  | Poor | 535 | 26.3 |

Note: ^a^ Values were presented as medians with interquartile ranges.

**Table S2. Associations of frequency of night shift, sleep duration, sleep quality with depressive, anxiety, and comorbid symptoms**

| **Variables** | | **Depressive symptoms** | **Anxiety symptoms** | **Comorbid symptoms** |
| --- | --- | --- | --- | --- |
|  |  | **OR (95% CI)** | | |
| Frequency of night shift (per month)^a^ | |  |  |  |
|  | ≤4 times | Ref | Ref | Ref |
|  | 5-9 times | **1.46 (1.20, 1.77)** | **1.29 (1.06, 1.58)** | **1.34 (1.09, 1.65)** |
|  | ≥10 times | **1.76 (1.31, 2.38)** | **1.48 (1.08, 2.03)** | **1.58 (1.18, 2.11)** |
| Sleep duration^b^ | |  |  |  |
|  | <7 hours | Ref | Ref | Ref |
|  | ≥7 hours | **0.56 (0.46, 0.70)** | **0.58 (0.48, 0.71)** | **0.56 (0.46, 0.70)** |
| Sleep quality^c^ | |  |  |  |
|  | Good | Ref | Ref | Ref |
|  | Fair | **2.82 (2.22, 3.60)** | **2.47 (1.97, 3.10)** | **2.69 (2.10, 3.45)** |
|  | Poor | **7.05 (5.20, 9.62)** | **4.58 (3.37, 6.26)** | **5.71 (4.24, 7.74)** |

Note: Bold values indicate statistical significance at p < 0.05.

Model^a^ was adjusted for age, sex, residence, education level, monthly income, smoking and drinking status, sleep duration, and sleep quality.

Model^b^ was adjusted for age, sex, residence, education level, monthly income, smoking and drinking status, and sleep quality.

Model^c^ was adjusted for age, sex, residence, education level, monthly income, smoking and drinking status, and sleep duration.

**Table S3. Associations of frequency of night shift with sleep duration and sleep quality**

| **Variables** | | **Sleep duration^b^** | **Sleep quality^c^** |
| --- | --- | --- | --- |
|  |  | **OR (95% CI)** | |
| Frequency of night shift (per month) | |  |  |
|  | ≤4 times | Ref | Ref |
|  | 5-9 times | 1.12 (0.89, 1.41) | **1.44 (1.13, 1.84)** |
|  | ≥10 times | 1.01 (0.72, 1.42) | **2.13 (1.46, 3.17)** |

Note: Bold values indicate statistical significance at p < 0.05.

Model^b^ was adjusted for age, sex, residence, education level, monthly income, smoking and drinking status, and sleep quality.

Model^c^ was adjusted for age, sex, residence, education level, monthly income, smoking and drinking status, and sleep duration.

**Table S4. Sensitivity analyses: associations between higher night-shift frequency and mental health outcomes after additional adjustment for work-related factors**

| Outcome | Model 1  OR (95% CI) | p | Model 2  OR (95% CI) | p |
| --- | --- | --- | --- | --- |
| Depressive symptoms | 1.50  (1.23, 1.84) | <0.001 | 1.54  (1.25, 1.90) | <0.001 |
| Anxiety symptoms | 1.33  (1.08, 1.64) | 0.006 | 1.35  (1.09, 1.68) | 0.006 |
| Comorbid symptoms | 1.46  (1.20, 1.78) | <0.001 | 1.50  (1.22, 1.85) | <0.001 |

Note: ORs compare nurses with higher night-shift frequency (>4 times/month) versus lower frequency (≤4 times/month). Model 1 = primary adjusted model (as in main text) plus additional adjustment for professional title and years of nursing experience. Model 2 = Model 1 plus additional adjustment for department assignment.

**Table S5. Sensitivity analysis: Prevalence ratios for associations between night shift frequency and mental health outcomes**

| Variables | Depressive symptoms  PR (95% CI) | Anxiety symptoms  PR (95% CI) | | Comorbid symptoms  PR (95% CI) |
| --- | --- | --- | --- | --- |
| Night shift frequency |  |  | |  |
| ≤4 times/month | Ref | Ref | Ref | |
| >4 times/month | 1.14 (1.05-1.24) | 1.07 (1.00-1.14) | 1.14 (1.04-1.25) | |

Note: PR, Prevalence Ratio; CI, Confidence Interval. Prevalence ratios were estimated using modified Poisson regression with robust variance. Log-binomial regression models did not converge; therefore, modified Poisson regression was used as a validated alternative approach for estimating prevalence ratios. Models were adjusted for age, sex, residence, education level, monthly income, smoking status, drinking status, sleep quality, and sleep duration
